# Mercury in India Rubber Dental Plates

**Published:** 1870-04

**Authors:** G. H. Perine


### ARTICLE VI.

### *Mercury in India Rubber Dental Plates.*

G. H. Perine. D. D. S.

**Writes as follows to the Editor of *Medical Gazette*.**

In the *Medical Gazette* of 22nd January is mentioned an important operation by Dr. Whitehead, of this city, and the mechanical substitution of the parts by Dr. Crane. The question is raised of the possible noxious effects of mercury from india-rubber dental plates; and as you ask the views of those having experience in the matter, I would present the following facts:

It has been now some fifteen years since this substance was first offered to the dental profession as a suitable base for artificial teeth.

For the first five or six years, it was but little employed by our best dentists, they having little confidence in its durability. It, however, slowly worked itself into favor with the profession and the public, until at the present time, eight-tenths, and probably a larger per cent. of all the artificial dentures made are mounted upon this base.

In the year 1863, it was estimated by Dr. B. W. Franklin, an eminent mechanical dentist of this city, from data kept by him and obtained from the manufacturers of porcelain teeth, and from the sale of dental rubber and materials, (in which he was then engaged,) that no less than one hundred thousand sets of artificial teeth were made upon vulcanite in the United States in that year. Since that time, its employment by the profession has become general, and the number of dentures on this base that are now being satisfactorily used, is inconceivably large. These facts are mentioned as arguments against the idea of any extensive injury resulting from the employment of this substance as a base for artificial teeth Pure vermilion, pure sulphur and properly prepared rubber (when carefully vulcanized), ought to be inert when placed in contact with living tissues. There is no acid known, which, when diluted to the strength of oral secretions in health or disease, acts upon vulcanite. Concentrated potash, chlorine, ammonia, and many active agents, have no action upon this substance. That some persons are more or less affected with hypertrophied conditions under all dental plates, is a fact cognizable by every practitioner of experience and observation; but that the vermilion in vulcanite dental plates has an agency in producing any extra unpleasant effects in any case coming under our own observation, we do not believe. It is true that much of the vermilion and sulphur of commerce are adulterated, and contaminated by poisonous substances, arsenic, red lead, oxide of zinc, and other substances.

Much of the dental rubber offered in the market, and used by some dentists. is compounded by parties having little if any knowledge of these adulterations. And yet notwithstanding this, tens of thousands of sets of teeth are annually made on vulcanite, and worn constantly, giving the utmost comfort and satisfaction. These teeth are in the mouths of patients of every grade of society, and in every possible physical condition. We were among the first to adopt this then) new base; we have watched its progress with interest, and have noted its effects; and we do not believe there has been a single case presenting itself anywhere, showing well defined evidence of the medicinal action of mercury.

That metallic mercury is present in the vulcanite rubber plate, can be demonstrated by burning a piece and collecting the products of combustion on glass or porcelain and rubbing the burnt mass on polished brass; the surface becomes immediately whitened. But the small amount of metallic mercury, or any reasonable amount of foreign or even poisonous matter with which the vermilion, or the sulphur, would be likely to be contaminated, could not exert their specific effects when enveloped in the insoluble vulcanite plate.

There are some persons so constituted that they cannot tolerate the constant presence of foreign substances in the mouth, without more or or less inconvenience; others present a constitutional predisposition to tumefaction of the mucous surfaces, rendering the wearing of any plate difficult, if not inadmissible. The superior adaptation of the vulcanite to the parts upon which the denture rests, especially with large air chambers, the plate being a poor conductor, is more liable to scald the parts, causing all the noxious results charged to the vermilion in the vulcanite plate. When we take into consideration the imperfect and crude manner in which artificial teeth are made, the little skill employed in their construction, the want of harmony in form and expression given, and the total absence of adaptibility in every essential requirement, the better part of the profession and the entire public ought to be thankful that so little permanent injury results. When the time shall come that the public will learn to discriminate between meritorious and skillful experience, and pretending, incompetent ignorance, both in the medical and dental professions, many of the evils now complained of will be traceable to legitimate causes, and find speedy and permanent relief.

---

